# Comment on Topaloglu et al. Machine Learning-Driven Lung Sound Analysis: Novel Methodology for Asthma Diagnosis. *Adv. Respir. Med.* 2025, *93*, 32

**DOI:** 10.3390/arm94020015

**Published:** 2026-02-28

**Authors:** Kazim Okan Dolu

**Affiliations:** Department of Pediatric Allergy and Immunology, Kanuni Sultan Süleyman Training and Research Hospital, Istanbul 34303, Türkiye; okandolu@gmail.com

I am writing regarding the article titled “Machine Learning-Driven Lung Sound Analysis: Novel Methodology for Asthma Diagnosis” published by Topaloglu et al. in *Advances in Respiratory Medicine* [1]. While the study presents an innovative application of machine learning to digital auscultation, several aspects of the methodology and reporting warrant clarification to ensure scientific traceability and reproducibility.

First, the manuscript reports the use of the “ICBHI 2017” dataset for external validation and describes an evaluation cohort including 47 asthma and 27 healthy subjects [1]. In contrast, the canonical ICBHI 2017 Respiratory Sound Database is described as comprising 126 participants and 920 recordings [2], and asthma is reported to be represented by only a single subject in commonly cited diagnostic distributions derived from this benchmark resource [3]. Moreover, the data availability statement in the manuscript refers to a Kaggle-hosted dataset titled “Asthma Detection Dataset Version 2 (ICBHI 2017)” [1,4]. Taken together, these statements create ambiguity regarding the provenance and exact version of the external validation data, which directly affects reproducibility and comparability with prior ICBHI-based studies.

Second, the reported performance metrics appear internally inconsistent. Table 4 reports an accuracy of 99.63% for the Narrow Neural Network model, whereas Figure 5 presents a ROC-AUC of 0.877 and an average precision of 0.868 for the same model [1]. If these metrics were derived from the same evaluation protocol, this discrepancy requires clarification regarding (i) which continuous scores were used for ROC/PR computation (probabilities vs. decision scores), (ii) the unit of analysis (segment-level vs. participant-level), and (iii) how 10-fold results were aggregated across folds (fold-wise averaging vs. pooled predictions) [1].

Third, the manuscript describes segmenting the recordings into 3 s windows [1]. Although participant-level stratified cross-validation is stated [1], the manuscript does not clearly specify whether preprocessing and feature selection steps (including ReliefF) were performed strictly within each training fold (i.e., as a fully nested pipeline). In segmentation-based studies, non-nested preprocessing or feature selection can lead to optimistically biased performance estimates and therefore should be explicitly documented, ideally with a pipeline schematic or code availability statement [1].

In summary, clarifying the exact external validation dataset and its relationship to the canonical ICBHI 2017 resource, as well as providing transparent details on metric computation and cross-validation workflow, would substantially strengthen the methodological transparency of this work.
